# Missense Variants in Nutrition-Related Genes: A Computational Study

**DOI:** 10.3390/ijms26199619

**Published:** 2025-10-02

**Authors:** Giovanni Maria De Filippis, Maria Monticelli, Bruno Hay Mele, Viola Calabrò

**Affiliations:** 1 Department of Electrical Engineering and Information Technology, University of Naples Federico II, Naples, via Claudio 21, 80125 Napoli, Italy; giovannimaria.defilippis@unina.it; 2 Biology Department, University of Naples Federico II, Complesso Universitario Monte Sant’Angelo, Via Cinthia, 80126 Napoli, Italy; maria.monticelli@unina.it (M.M.); viola.calabro@unina.it (V.C.); 3 Institute of Biomolecular Chemistry ICB, Consiglio Nazionale delle Ricerche (CNR), Via Campi Flegrei 34, 80078 Pozzuoli, Italy

**Keywords:** nutrigenetics, PPI networks, variant effect prediction, data augmentation, MeSH ontology

## Abstract

Genetic variants in nutrition-related genes exhibit variable functional consequences; however, systematic characterization across different nutritional domains remains limited. This highlights the need for detailed exploration of variant distribution and functional effects across nutritional gene categories. Therefore, the main objective of this computational study is to delve deeper into the distribution and functional impact of missense variants in nutrition-related genes. We analyzed Genetic polymoRphism variants using Personalized Medicine (GRPM) dataset, focusing on ten groups of nutrition-related genes. Missense variants were characterized using ProtVar for functional/structural impact, Pharos for functional classification, network analysis for pathway identification, and Gene Ontology enrichment for biological process annotation. The analysis of 63,581 Single Nucleotide Polymorphisms (SNP) revealed 27,683 missense variants across 1589 genes. Food intolerance (0.23) and food allergy (0.15) groups showed the highest missense/SNP ratio, while obesity-related genes showed the lowest (0.04). Enzymes predominated in xenobiotic and vitamin metabolism groups, while G-protein-coupled receptors were enriched in eating behavior genes. The vitamin metabolism group had the highest proportion of pathogenic variants. Network analysis identified apolipoproteins as central hubs in metabolic groups and inflammatory proteins in allergy-related groups. These findings offer insights into personalized nutrition approaches and underscore the utility of computational variant analysis in elucidating gene-diet interactions.

## 1. Introduction

Understanding the relationship between genetic variation and nutrition-related phenotypes is fundamental to advancing personalized medicine and nutrition. Genetic variants affecting nutritional metabolism, food tolerance, and dietary response exhibit highly variable functional consequences, yet systematic characterization of these effects across different nutritional domains remains limited.

The evolutionary perspective provides crucial insights into nutrition-related genetic variation. Many variants implicated in food intolerance, allergy, and metabolic disorders represent common polymorphisms shaped by historical selective pressures rather than recent deleterious mutations. This observation raises a fundamental question: why do disease-associated variants persist at appreciable frequencies in human populations? Evidence suggests these variants often represent evolutionary trade-offs, where historical advantages balanced current risks. For example, alleles in the APOL1 gene, which confer resistance to *Trypanosoma brucei* infection, also predispose carriers to kidney disease [1]. Similarly, immune-related genes such as HLA, IL4, or chemokine receptors (CCR3, CXCR5), frequently implicated in food allergies, are known to be subject to balancing selection driven by historical pathogen exposures [2]. In these cases, variants that may today contribute to allergy or autoimmunity could have conferred survival benefits in infectious environments, explaining their persistence and relatively high frequencies [3]. This pattern contrasts with the evolutionary dynamics of rare, highly deleterious variants—such as those causing familial hypercholesterolemia—which are efficiently removed from the population by purifying selection [4]. As such, a broader principle emerges: variants associated with immune-mediated food responses or micronutrient handling are often evolutionarily tolerated, either because they posed minimal reproductive cost or because they were once adaptive. Mechanistically, these variants often act not by abolishing protein function outright, but by subtly modifying protein stability, folding, or regulation.

Missense mutations in enzymes involved in vitamin metabolism, for example, frequently impair conformational stability rather than active site chemistry [5,6]. Structural studies and protein modeling have shown that such mutations can destabilize the native fold, reduce thermal tolerance, or impair subunit interactions—leading to partial loss-of-function. This insight has guided the development of pharmacological chaperones for some inborn errors of metabolism, illustrating the therapeutic relevance of structural interpretation [7,8]. Likewise, G-protein-coupled receptors (GPCRs)—which mediate taste perception, gut–brain hunger signaling, and food reward—harbor common variants affecting individual differences in dietary preference and metabolic response. Taste receptor polymorphisms (e.g., T1R2/T1R3, TAS2R) influence sweetness or bitterness perception, while genetic variation in dopaminergic or opioid GPCRs can modulate the hedonic response to food, potentially contributing to compulsive eating or obesity [9,10].

The integration of omics technologies has transformed our understanding of complex biological systems, enabling large-scale investigations across genomics, proteomics, and metabolomics [11,12]. In the context of personalized nutrition, genetic data are key to uncovering how individual variability influences dietary responses and health outcomes. Progress in this field increasingly depends on the availability of curated, interoperable datasets that bridge genetics and nutrition. One such resource is GRPM (Gene-RsID-PMID-MeSH), a publicly available dataset of genetic polymorphisms associated with nutrition-related traits (https://zenodo.org/records/14052302, accessed on 31 March 2025), constructed through the integration of multiple data sources and structured using the MeSH ontology [13]. Its standardized, ontology-driven design facilitates systematic analysis of gene–diet interactions and supports the development of targeted interventions in nutrigenetics and personalized nutrition.

Despite this progress, a comprehensive understanding of how missense variants impact nutrition-related genes remains limited. We hypothesize that genes involved in different nutritional processes exhibit distinct patterns of evolutionary constraint and functional variation, reflecting their varying biological importance and the selective pressures they have experienced. Therefore, the main objective of this computational study is to delve deeper into the distribution and functional impact of missense variants in nutrition-related genes.

**Figure 1:**
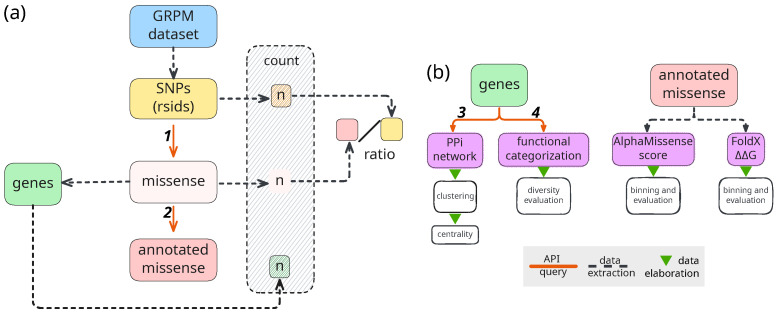
Schematic representation of the pipeline. (**a**) Data retrieval and extraction; (**b**) data analysis. Dashed lines mark data produced through extraction processes (i.e., subsetting and filtering files), green triangles mark data elaboration (analysis and visualization), solid orange lines mark API queries to the following services: 1. BioMart, 2. ProtVar, 3. Pharos, 4. StringDb. All services are referenced in the text. Abbreviations: GRPM: source dataset (**G**ene-**R**sID-**P**MID-**M**eSH) [13]; SNPs: Single Nucleotide Polymorphisms; PPI: protein–protein interaction.

We developed a comprehensive computational pipeline (Figure 1) that integrates multiple data sources and analytical approaches. Our specific objectives were to (1) quantify the distribution of missense variants across different nutrition-related gene groups; (2) assess the functional and structural impact of these variants using computational prediction tools; (3) identify functional patterns and protein classes enriched in each category; and (4) characterize protein interaction networks and biological pathways associated with each gene group.

## 2. Results

### 2.1. Missense/SNP Ratio and Functional Constraint

Our analysis included 63,581 Single Nucleotide Polymorphisms from the GRPM dataset, of which approximately 40% were successfully mapped to ProtVar for detailed structural and functional predictions. This mapping yielded 27,683 missense variants across 1589 unique genes distributed among the ten defined nutritional categories: (1) Cardiovascular Health and Lipid Metabolism (CHLM), (2) Diabetes Mellitus Type II and Metabolic Syndrome (DM&MS), (3) Diet-induced Oxidative Stress (DiOS), (4) Eating Behavior and Taste Sensation (EBTS), (5) Food Allergies (FAs), (6) Food Intolerances (FIs), (7) General Nutrition (GN), (8) Obesity, Weight Control and Compulsive Eating (OWG&CE), (9) Vitamin and Micronutrient Metabolism and Deficiency-Related Diseases (VMM&DRD), and (10) Xenobiotic Metabolism (XM) (Table 1). The complete list of genes containing the rsIDs analyzed is available as Supplementary Material (File S1). A comprehensive description of the ten nutritional categories is available in [13].

The distribution of variants across the ten nutritional categories revealed significant differences in missense/SNP ratios, ranging from 0.04 to 0.23 (Table 1). Food Intolerances showed the highest ratio (0.23), followed by Food Allergies (FAs, 0.15), suggesting these gene groups tolerate more missense variation. Conversely, Obesity, Weight Control and Compulsive Eating exhibited the lowest ratio (0.04), indicating stronger evolutionary constraints.

### 2.2. Variant Composition Across Nutritional Gene Groups

**Figure 2:**
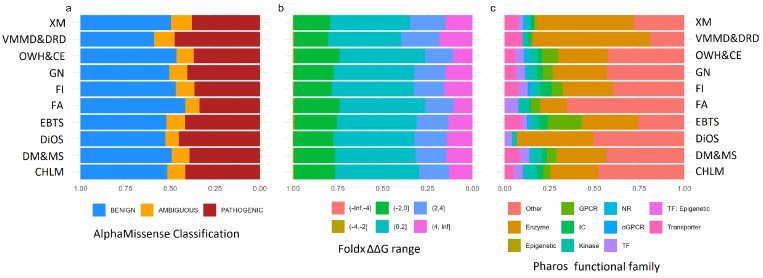
(**a**) Pathogenicity score distribution across nutrigentic topics. AlphaMissense classification, 0 to 0.34 means benign, 0.34 to 0.564 uncertain, 0.564 to 1 likely pathogenic [14]. (**b**) Energy perturbation (FoldX |ΔΔ| G) distribution, values > 2.0 kcal/mol and 4.0 kcal/mol are considered destabilizing and severely destabilizing, respectively [15]. (**c**) Functional family assignment distribution (Pharos annotation) within each nutrigenetic topic. Abbreviations: Cardiovascular Health and Lipid Metabolism (CHLM), Diabetes Mellitus Type II and Metabolic Syndrome (DM&MS), Diet-induced Oxidative Stress (DiOS), Eating Behavior and Taste Sensation (EBTS), Food Allergies (FAs), Food Intolerances (FI), General Nutrition (GN), Obesity, Weight Control and Compulsive Eating (OWG&CE), Vitamin and Micronutrient Metabolism and Deficiency-Related Diseases (VMM&DRD), and Xenobiotic Metabolism (XM).

The analysis of the relative distribution of functional families associated with missense variants revealed a predominance of enzymes in the Xenobiotic Metabolism and Vitamin and Micronutrients Metabolism and Deficiency-Related Diseases groups. The Eating Behavior and Taste Sensation group showed a clear prevalence of GPCRs, while an unexpected predominance of “other” protein classes was observed in the Food Allergies group (Figure 2).

An evaluation of the distribution of variants based on predicted destabilization effects showed a generally homogeneous pattern between groups, with a slight increase in highly destabilizing mutations within the Vitamin and Micronutrient Metabolism and Deficiency-Related Diseases category (Figure 2). Very few highly stabilizing mutations seem to be predicted. This same group also showed the highest number of variants classified as “pathogenic” (Figure 2). The number of Variants of Uncertain Significance (VUS) was extremely small in all groups.

### 2.3. Network and Gene Ontology Analysis

**Figure 3:**
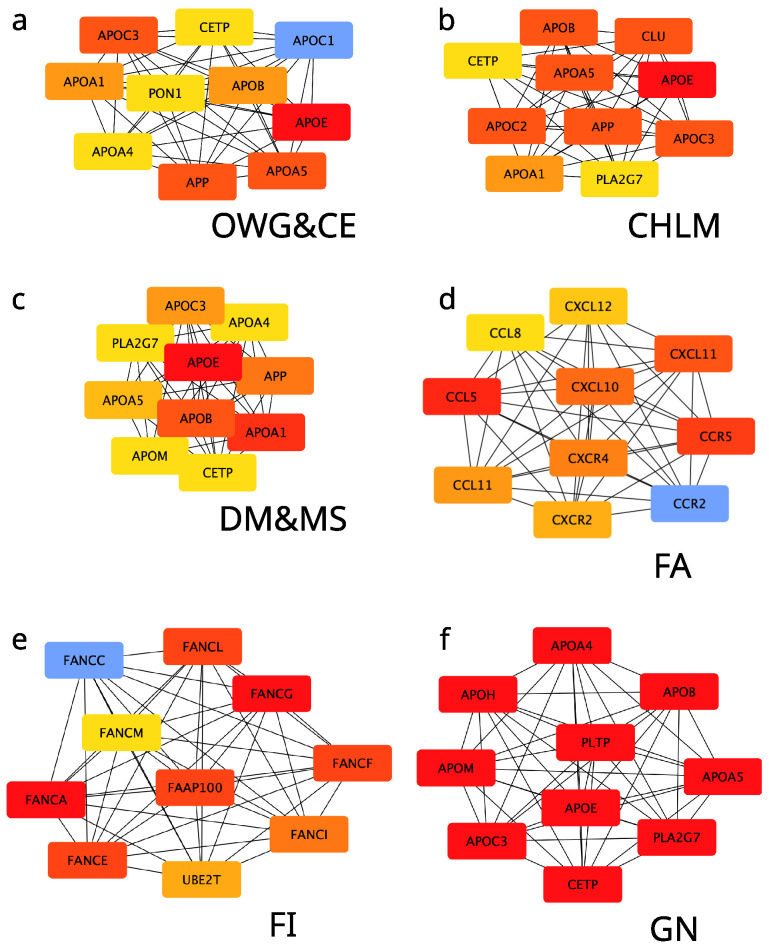
Top 10 hub genes identified on the richest networks by the CytoHubba plugin in Cytoscape, using the Maximal Clique Centrality algorithm. These hub genes are highly connected, essential nodes within a scale-free PPI network, integrating diverse functional partners and linking multiple network components. Red intensity marks the importance, going from blue (lowest) to full red (highest). Group acronyms are as follows: (**a**) OWG&CE Obesity—Weight Control and Compulsive Eating; (**b**) CHLM—Cardiovascular Health and Lipid Metabolism; (**c**) DM&MS—Diabetes Mellitus Type II and Metabolic Syndrome; (**d**) FAs—Food Allergies; (**e**) FIs—Food Intolerances; (**f**) GN—General Nutrition.

Protein–protein interaction (PPi) network analysis revealed that four out of ten groups are mainly composed of singletons and form few, scarcely populated clusters, while the other six produced dense clusters. Identification of central nodes (hubs) for genes implied in each group revealed interesting results (Figure 3). Apolipoproteins (APOs) were identified as central nodes in the non-food-related groups: Obesity, Weight Control and Compulsive Eating (panel a); Cardiovascular Health and Lipid Metabolism (panel b); Diabetes Mellitus Type II and Metabolic Syndrome (panel c); and General Nutrition (panel d). Notably, the General Nutrition group, broad yet less specific, still highlighted APOs as key players, emerging as a cross-category encompassing diverse aspects of other groups. Food allergies were mainly related to inflammatory state proteins (panel d), while the Food Intolerances group was surprisingly characterized by the centrality of Fanconi anemia (FA) proteins. The four out of ten groups comprising a low amount of proteins and showing many isolated nodes or pairs did not lead to a hub search (Supplementary Figure S1).

Gene Ontology (GO) analysis revealed overlapping patterns, as expected from network analysis (Additional Table A1). Cellular components (CC) GO as significantly enriched by far in extracellular context (i.e., extracellular space, extracellular region, plasma membrane region, external side of plasma membrane, and vescicle) in all the analyzed groups. Most of the biological processes (BPs) could be subsets of three major groups: (i) involvement in homeostasis (i.e., cellular homeostasis and homeostatic process); (ii) specialized metabolism (modified amino acid metabolic process; nitric oxide synthase biosynthetic process; organic acid metabolic process; secondary alcohol metabolic process; small molecule metabolic process; and vitamin metabolic process); and (iii) response to external stimuli (i.e., response to xenobiotic stimulus, response to chemical, response to interleukine-15, response to oxygen-containing compound, response to stress, and response to toxic substance). Interestingly, molecular functions (MFs) were largely related to molecule binding (cytokine binding, cytokine receptor binding, hormone binding, identical protein binding, lipid binding, signaling receptor binding, small molecule binding, tetrapyrrole binding, and vitamin binding). Besides this, a significant involvement of oxidoreductase and antioxidant activity was observed.

## 3. Discussion

This study provides an integrated, large-scale computational examination of missense variants in nutrition-related genes, focusing on evolutionary constraint and functional impact across ten systematically defined nutritional categories. These categories balance breadth (general nutrition, metabolic disease) with specificity (food intolerances, eating behavior, oxidative stress), ensuring clinical and mechanistic relevance (see [13] for methodological detail).

At the outset, this study contributes in three complementary ways: (1) it presents the first large-scale comparative analysis of missense variation across ten categories of nutrition-related genes, with systematic evaluation of functional consequences; (2) it integrates multiple computational pipelines (structural/functional predictions, network centrality, enrichment analyses) to generate a comprehensive, multi-level variant impact map; and (3) it offers a bioinformatics resource that can support personalized nutrition, disease risk stratification, and experimental follow-up, aligning with precision medicine initiatives. Together, these contributions establish novelty, scope, and translational relevance for the ensuing interpretation of constraint and function.

Our comparative findings reveal stratified tolerance to missense variation: genes implicated in food allergies and intolerances display higher missense/SNP ratios (0.15–0.23), consistent with relaxed constraint or diversifying pressures, whereas genes central to core metabolic homeostasis (e.g., obesity and cardiovascular-related pathways) show markedly lower ratios (0.04–0.08), indicative of stronger purifying selection. This gradient supports the hypothesis that distinct nutritional gene subclasses have been shaped by heterogeneous selective regimes across human evolution and provides a functional scaffold for prioritizing variants for experimental or clinical follow-up.

### 3.1. Variant Classification and Evolutionary Insights

The number of VUS was extremely small in all groups, highlighting the effectiveness of the GRPM method in variant classification. Such a low prevalence of VUS underscores the reliability and precision of the GRPM approach, as it successfully categorizes variants with clear effects, minimizing ambiguity in classification.

Interestingly, in our analysis, gene groups related to food allergies and food intolerances exhibited the highest missense-to-SNP ratios (0.15 and 0.23, respectively), consistent with previous findings suggesting that immune and digestive system genes are more tolerant of common variation. This likely reflects historical balancing selection or environmental adaptation [3]. In contrast, genes associated with cardiometabolic traits—such as cardiovascular disease and type 2 diabetes—showed lower missense-to-SNP ratios, suggesting stronger evolutionary constraints. The severe consequences of dysfunction in these pathways likely lead to stronger purifying selection, reducing the prevalence of deleterious variants in the population [4]. Notably, missense variants implicated in food allergies were generally not shared with other disease categories, supporting their relative specificity. The especially low missense/SNP ratio observed in the “Obesity, Weight Control, and Compulsive Eating” category further underscores the influence of selective pressures in shaping gene variant distributions.

The high frequency of missense variants in genes related to food intolerance and allergy likely reflects reduced reproductive penalties or even potential evolutionary advantages, such as improved pathogen resistance or dietary adaptability. Conversely, variants causing severe cardiometabolic disease have remained rare due to the fitness costs they impose.

These findings underscore the non-uniform distribution of variant tolerance across nutrition-related pathways and suggest that genes involved in food perception and immune interaction have evolved under different selective regimes than those governing core metabolic and cardiovascular processes.

### 3.2. Inflammation and Cardiovascular Risks from Food Allergies

While most food allergy-related variants appear to be relatively benign, our analysis found that food intolerance-associated variants may intersect with pathways implicated in cardiovascular disease. Food allergies are mediated by abnormal immune responses to specific dietary proteins. Chronic exposure to allergens can lead to persistent inflammation, in which the CCL and CXCR chemokine families play a central role [16]. This prolonged inflammatory state may extend systemically, influencing cardiovascular health.

Chronic inflammation is a known driver of atherosclerosis and endothelial dysfunction, with cytokines such as IL-1β, IL-6, and TNF-α, and chemokines like CCL2/MCP-1 promoting immune cell infiltration and plaque formation [17]. A 2017 review by Castan et al. highlighted the role of chemokine networks in orchestrating immune responses in food allergy, asthma, and atopic dermatitis [16]. A 2024 longitudinal study (NHANES and MESA cohorts) found that individuals sensitized to common foods exhibited significantly increased cardiovascular mortality, particularly those with IgE sensitization to milk (HR 2.0–3.8) [18]. These findings support a systemic model of food allergy pathophysiology, wherein sustained immune activation contributes to vascular inflammation and cardiovascular risk.

### 3.3. Enzymes, GPCRs, and Eating Behavior

In our dataset, enzymes were prominently represented in the category of vitamin and micronutrient metabolism. Missense mutations in this group predominantly caused mild to severe enzyme destabilization, consistent with prior mechanistic studies. GPCRs were highly enriched in categories related to taste sensation and eating behavior, reflecting their established role in detecting food-derived chemical cues and modulating physiological and emotional responses. T1R and T2R GPCRs mediate sweet, umami, and bitter taste perception, influencing food preferences and aversions [9]. Taste receptors are expressed in several extra-oral tissues, where they play important physiological roles [19], including the respiratory system [20], the gastrointestinal tract [21], and reproductive tissues, especially testis [22]. Beyond sensory input, other GPCRs regulate hunger and satiety through pathways involving ghrelin and leptin, and also participate in brain reward systems, such as dopamine signaling [23,24,25,26]. Studies such as the one from Lee and co-workers have shown that sweet taste receptors (T1R2/T1R3) integrate nutrient sensing with behavioral outputs [27]. The role of dopamine D1 and D2 receptors in the nucleus accumbens and striatum further links eating behavior with reward and reinforcement mechanisms [28,29,30]. Notably, research by Johnson and Kenny demonstrated that rats exposed to hyperpalatable diets developed compulsive eating and reward deficits akin to addiction, accompanied by downregulation of D2 receptors [31]. Similar patterns have been observed in human neuroimaging studies, where reduced striatal D2 receptor availability correlates with obesity and altered reward sensitivity. Given these broader functions, taste receptor genes may be under stronger negative selection than expected from their role in taste or food intolerance alone.

### 3.4. Fanconi Anemia Proteins and Food Intolerance

One of the most intriguing findings of our analysis is the central role of Fanconi anemia (FA) proteins in food intolerance—and to a lesser extent, food allergy. FA proteins are primarily known for their role in DNA repair and genome stability. In patients with Fanconi anemia, poor digestive tolerance has been documented and may underlie the observed association with food intolerance [32]. Moreover, the underlying defect in the DNA repair system is known to predispose individuals to different cancer types, including gastrointestinal malignancies [33,34,35]. Although not directly linked to dietary responses, we hypothesize that exposure to intolerance-inducing foods may cause cellular stress or damage, triggering DNA repair pathways. UBE2T, which mono-ubiquitinates FANCD2, is a key activator of the FA pathway, facilitating the repair of DNA interstrand crosslinks. Furthermore, intestinal barrier dysfunction in food intolerance may involve epithelial stress responses where FA proteins contribute to cell maintenance. It is also possible that FA proteins participate in modulating immune function, given the immune component of food intolerance. While the precise mechanism remains unclear, these observations warrant further investigation into the role of FA genes in gut-immune homeostasis and food-related immune responses.

### 3.5. Role of Apolipoproteins in Metabolic Health

Lastly, apolipoproteins (APOs) emerged as critical hubs linking obesity, cardiovascular disease, and general nutrition. APO variants influence lipid metabolism, impacting how lipids are absorbed, transported, and cleared from the bloodstream [36]. Due to their central metabolic roles, APO genes exhibit high natural variability, likely shaped by evolutionary pressures such as dietary shifts or infectious disease exposure. For instance, the ApoE gene has three common alleles (ε2,ε3,ε4), each associated with different disease risks [37]. While the ε4 allele increases cardiovascular and Alzheimer’s disease risk [38,39], it may have offered adaptive benefits in certain ancestral environments [40]. Similarly, the APOL1 risk alleles—common in African populations—confer resistance to Trypanosoma infection at the cost of increased kidney disease risk [41]. These examples illustrate the trade-offs inherent in human genetic diversity. Personalized approaches, such as tailoring diets based on APOE genotype, highlight the potential clinical utility of understanding this variability. Moreover, the rarity of highly pathogenic alleles in core metabolic genes (e.g., LDLR mutations in familial hypercholesterolemia) reinforces the principle that life-threatening variants are tightly constrained by natural selection.

### 3.6. Implications for Precision Nutrition and Personalized Medicine

Together, these findings illustrate the complex interplay of genetic variation, evolutionary pressure, and disease risk across nutritionally relevant genes. Genes involved in food perception, immune interaction, and metabolism exhibit distinct patterns of variation shaped by both historical and modern environments. This has significant implications for precision nutrition and personalized medicine.

Our key findings demonstrate that (1) genes involved in food allergies and intolerances exhibit higher tolerance to missense variation, likely reflecting historical balancing selection; (2) functional protein classes are differentially enriched across categories, with enzymes predominating in metabolic groups and GPCRs in sensory/behavioral groups; and (3) network analysis identifies apolipoproteins as central hubs in multiple metabolic categories. These findings contribute to our understanding of the functional landscape of nutrition-related genetic variation and provide a foundation for developing more precise and effective personalized nutrition interventions. The differential patterns of variant tolerance across nutritional categories suggest that genetic testing strategies should be tailored to the specific nutritional domain of interest. For instance, screening for variants in food allergy and intolerance genes may require different interpretation criteria than variants in core metabolic pathways. The identification of apolipoproteins as central network hubs across multiple metabolic categories highlights their potential as targets for nutrigenetic interventions.

### 3.7. Limitations and Future Directions

While our study provides valuable insights into nutrition-related genetic variation, several limitations should be acknowledged. The GRPM dataset aggregates genetic variants identified across diverse studies and populations, thereby capturing stable genetic polymorphisms that are not influenced by age or sex stratification and are reported as. However, interpreting their functional and clinical implications in specific populations may require consideration of demographic factors. The analysis was limited to variants with available rsIDs mappable to ProtVar, which may introduce selection bias. Future studies should incorporate population-specific variant databases and experimental validation of computational predictions. Additionally, the functional consequences of variants should be validated through in vitro and in vivo studies to confirm the computational predictions presented here.

## 4. Materials and Methods

### 4.1. Study Design and Data Source

This computational study employed a cross-sectional analysis of genetic variants using publicly available databases. We utilized the GRPM dataset, a curated collection of genetic polymorphisms associated with nutrition-related traits [13]. This dataset integrates multiple data sources and is structured using the Medical Subject Headings (MeSH) ontology to facilitate systematic analysis of gene–diet interactions.

From the GRPM dataset, we selected variants annotated with reference SNP cluster ID (rsID) numbers for comprehensive analysis. The dataset categorizes genes into ten nutrition-related groups as defined in [13]: (1) Cardiovascular Health and Lipid Metabolism, (2) Diabetes Mellitus Type II and Metabolic Syndrome, (3) Diet-induced Oxidative Stress, (4) Eating Behavior and Taste Sensation, (5) Food Allergies (FAs), (6) Food Intolerances, (7) General Nutrition, (8) Obesity, Weight Control and Compulsive Eating, (9) Vitamin and Micronutrient Metabolism and Deficiency-Related Diseases, and (10) Xenobiotic Metabolism. These categories reflect clinically and nutritionally relevant pathways, including nutrition physiology, nutrition-related diseases, disease prevention through diet, and eating behavior. For more details, we refer the reader to [13].

### 4.2. Data Retrieval and Annotation

We wrote an R script to download and unzip the zenodo record associated with the core dataset (https://zenodo.org/records/14052302, accessed on 31 March 2025). The dataset was imported into R using the read_parquet() function. From these datasets we extracted the list of unique reference SNP identifiers (rsids), which serve as variant makers. To annotate these SNPs, we utilized the Ensembl BioMart interface via the biomaRt package. We queried the hsapiens_snp dataset within the Ensembl SNP mart to retrieve variant-level annotations including rsid, chromosomal position, and alleles. We focused on variants for which the consequence_type_tv was “missense_variant” (i.e., variants that have the potential to alter protein structure/function through amino acid substitution). We enriched the SNP list by manually uploading the rsid list to the ProtVar web platform (https://www.ebi.ac.uk/ProtVar/, accessed on 31 March 2025) [42], which provides comprehensive variant effect predictions including structural and functional annotations. Using ProtVar’s “Mapping with Annotations” functionality, we generated an annotated dataset that includes a variety of functional and structural annotations relevant to the protein context of each variant. Finally, to facilitate subsequent integration and processing, the ProtVar-annotated CSV file was converted to Parquet format and saved for subsequent analyses.

### 4.3. Functional Classification and Pathway Analysis

Functional categorization of proteins containing missense variants was performed using Pharos version 3.19 (https://pharos.nih.gov/, accessed on 31 March 2025), which classifies proteins based on their therapeutic relevance and functional properties. A custom GraphQL interface was used to query Pharos based on gene symbols. The data retrieved contained functional family annotations, including enzymes, GPCRs, ion channels, kinases, transcription factors, and other less-characterized categories. These classifications were used to stratify the variants based on their likely biological roles and potential therapeutic relevance.

### 4.4. Structural Impact Prediction

Structural impact of missense variants was assessed using complementary metrics retrieved from the protvar query: AlphaMissense pathogenicity scores and FoldX ΔΔG energy perturbation predictions. In the first case (alphamissense), variants were classified as pathogenic, likely pathogenic, VUS, likely benign, or benign based on AlphaMissense scores. Alphamissense is based on a deep learning model trained on evolutionary and clinical variant data [14]. As for FoldX, it estimates the changes in Gibbs free energy caused by the substitution, where higher-magnitude changes denote protein destabilization. Analyses were performed separately, and in both cases, variants were retained only if the specific score was available. In both cases, values were binned to reflect increasing levels of perturbation (pathogenicity or stability).

### 4.5. Network Analysis

Protein–protein interaction networks were constructed using the STRING database (https://string-db.org/, accessed on 31 March 2025) to identify hub proteins and functional clusters within each gene group. Network analysis was performed using Cytoscape software (version 3.10.4) with the CytoHubba plugin, employing the MCC algorithm to identify highly connected, essential nodes within scale-free networks. We used Cytoscape [43] to further investigate the genes associated with each topic. The STRINGdb plugin [44] was used to explore high confidence (Score > 0.7) protein–protein physical interaction networks for each of the ten nutrigenetic fields. To identify central proteins, we applied the CytoHubba plugin [45] within CytoScape, using the MCC algorithm. The top ten ranked hub proteins were selected for further interpretation. Maximal Clique Centrality ranks a node by how many fully connected “cliques” it belongs to—boosting the score further when those cliques are large—so the nodes sitting in the center of numerous, tightly knit groups stand out. A clique is a group where every member is directly connected to every other member.

### 4.6. Gene Ontology Analysis

To further explore the biological implications of the variant-bearing genes, we performed Gene Ontology (GO) enrichment analysis. This included overrepresentation testing for biological process, molecular function, and cellular component terms. GO terms enriched within each topical gene group helped highlight common pathways, cellular compartments, or enzymatic functions that may be particularly relevant to nutrient response or metabolism.

### 4.7. Statistical Analysis

For each gene group, we calculated the missense/SNP ratio as an indicator of evolutionary constraint and functional tolerance. We quantified the total number of SNPs, number of missense variants, and number of affected genes for each category. These metrics were compiled into a summary table and visualized for comparative interpretation. All computational analyses were performed using standard bioinformatics tools and databases as described above.

All code, including dataset handling, annotation parsing, and visualization routines, was implemented in R using a combination of tidyverse [46], jsonlite [47], arrow [48], httr [49] and GraphQL APIs (for Pharos). Visualization for non-network-related data was performed using ggplot2. R scripts are available as Supplementary Material (File S2) and in the project repository (https://github.com/johndef64/grpm_missense, accessed on 31 March 2025).

All queries were performed throughout the month of March 2025.

## 5. Conclusions

This comprehensive computational analysis of missense variants in nutrition-related genes reveals distinct evolutionary and functional patterns across different nutritional categories. Genes involved in food allergies and intolerances show increased tolerance to missense variation, and functional protein classes are differentially enriched across categories. Enzymes predominate in metabolic groups, and G protein-coupled receptors in sensory/behavioral groups. In our study, network analysis identifies apolipoproteins as central hubs in multiple metabolic categories.

The computational framework presented here demonstrates the value of integrating multiple data sources and prediction tools for comprehensive variant analysis in nutrigenetics research.

## Figures and Tables

**Table 1:** GRPM variants with an rsid found in ProtVar, grouped by topics. **SNP Count** refers to the total number of SNPs with rsid found in the GRMP dataset for each group. **Missense Count** refers to the number of variants having the “missense” value under the consequence key attribute in the Ensembl BiomaRt; **Missense Gene Count** is the number of genes containing at least one missense variant; The **Missense/SNP Ratio** is the ratio between the Missense and SNP counts. The group acronyms are as follows: CHLM—Cardiovascular Health and Lipid Metabolism; DM&MS—Diabetes Mellitus Type II and Metabolic Syndrome; DiOS—Diet-induced Oxidative Stress; EBTS—Eating Behavior and Taste Sensation; FAs—Food Allergies; FI—Food Intolerances; GN—General Nutrition; OWG&CE Obesity, Weight Control and Compulsive Eating; VMM&DRD—Vitamin and Micronutrient Metabolism and Deficiency-Related Diseases; XM—Xenobiotic Metabolism.

Group	SNP Count	Missense Count	Missense Gene Count	Missense/SNP Ratio
CHLM	365,243	28,294	938	0.08
DM&MS	215,861	11,752	524	0.05
DiOS	34,510	1686	73	0.05
EBTS	28,296	2406	192	0.09
FA	27,280	4227	371	0.15
FI	16,665	3766	255	0.23
GN	268,301	14,840	623	0.06
OWG&CE	118,198	4661	282	0.04
VMM&DRD	34,600	2457	90	0.07
XM	62,295	5878	185	0.09

## Data Availability

The original contributions presented in this study are included in the article/Supplementary Materials. Further inquiries can be directed to the corresponding author(s).

## Supplementary Materials

The following supporting information can be downloaded at https://www.mdpi.com/article/10.3390/ijms26199619/s1.

## Appendix A

**Table A1:** Gene ontology analysis (top three terms per each category within each nutrigenetic group). Group abbreviation as per (Table 1).

Group	GO Category	GO Term ID	GO Term Name	*p*-Value
CHLM	GO:MF	GO:0005102	Signaling receptor binding	$5.79\times 10^{-51}$
CHLM	GO:MF	GO:0042802	Identical protein binding	$3.125\times 10^{-49}$
CHLM	GO:MF	GO:0097367	Carbohydrate derivative binding	$3.02\times 10^{-23}$
CHLM	GO:BP	GO:1901700	Response to oxygen-containing compound	$1.32\times 10^{-137}$
CHLM	GO:BP	GO:0044281	Small molecule metabolic process	$1.06\times 10^{-67}$
CHLM	GO:BP	GO:0030198	Extracellular matrix organization	$1.79\times 10^{-23}$
CHLM	GO:CC	GO:0071944	Cell periphery	$6.39\times 10^{-57}$
CHLM	GO:CC	GO:0034358	Plasma lipoprotein particle	$4.02\times 10^{-26}$
CHLM	GO:CC	GO:0005739	Mitochondrion	$3.99\times 10^{-19}$
DM&MS	GO:MF	GO:0005102	Signaling Receptor Binding	$2.77\times 10^{-25}$
DM&MS	GO:MF	GO:0008289	Lipid binding	$3.40\times 10^{-19}$
DM&MS	GO:MF	GO:0042802	Identical Protein Binding	$5.61\times 10^{-19}$
DM&MS	GO:BP	GO:1901700	Response to oxygen-containing compound	$2.07\times 10^{-101}$
DM&MS	GO:BP	GO:0042592	Homeostatic process	$3.26\times 10^{-97}$
DM&MS	GO:BP	GO:0010876	Lipid localization	$6.7\times 10^{-68}$
DM&MS	GO:CC	GO:0005615	Extracellular space	$1.40\times 10^{-36}$
DM&MS	GO:CC	GO:0005783	Endoplasmic reticulum	$1.75\times 10^{-26}$
DM&MS	GO:CC	GO:0045177	Apical part of cell	$1.87\times 10^{-25}$
GN	GO:MF	GO:0042802	Identical protein bidning	$3.68\times 10^{-29}$
GN	GO:MF	GO:0005102	Signaling receptor binding	$1.04\times 10^{-27}$
GN	GO:MF	GO:0046906	Tetrapyrrole binding	$2.51\times 10^{-19}$
GN	GO:BP	GO:0042221	Response to chemical	$9.83\times 10^{-106}$
GN	GO:BP	GO:0003013	Circulatory system process	$5.89\times 10^{-60}$
GN	GO:BP	GO:0019216	Regulation of lipid metabolic process	$7.08\times 10^{-46}$
GN	GO:CC	GO:0005615	Extracellular space	$1.05\times 10^{-35}$
GN	GO:CC	GO:0012505	Endomembrane system	$1.88\times 10^{-32}$
GN	GO:CC	GO:0045177	Apical part of cell	$1.39\times 10^{-23}$
DiOS	GO:MF	GO:0016209	Antioxidant activity	$4.015\times 10^{-23}$
DiOS	GO:MF	GO:0016491	Oxidoreductase activity	$1.66\times 10^{-15}$
DiOS	GO:BP	GO:0009636	Response to toxic substance	$1.85\times 10^{-29}$
DiOS	GO:BP	GO:0006950	Response to stress	$6.71\times 10^{-29}$
DiOS	GO:BP	GO:0019725	Cellular homeostasis	$1.52\times 10^{-14}$
DiOS	GO:CC	GO:0005615	Extracellular space	$2.1\times 10^{-13}$
EBTS	GO:MF	GO:0008527	Taste receptor activity	$1.10\times 10^{-28}$
EBTS	GO:MF	GO:0004930	G protein-coupled receptor activity	$3.55\times 10^{-14}$
EBTS	GO:BP	GO:0044281	Small molecule metabolic process	$2.31\times 10^{-45}$
EBTS	GO:BP	GO:0042221	Response to chemical	$8.87\times 10^{-44}$
EBTS	GO:BP	GO:0007631	Feeding behavior	$7.97\times 10^{-11}$
EBTS	GO:CC	GO:0071944	Cell periphery	$1.715\times 10^{-12}$
XM	GO:MF	GO:0016491	Oxidoreductase activity	$2.95\times 10^{-47}$
XM	GO:MF	GO:0046906	Tetrapyrrole binding	$8.09\times 10^{-29}$
XM	GO:MF	GO:0036094	Small molecule binding	$4.49\times 10^{-15}$
XM	GO:BP	GO:0009410	Responde to xenobiotic stimulus	$1.32\times 10^{-71}$
XM	GO:BP	GO:0044281	Small molecule metabolic process	$2.78\times 10^{-64}$
XM	GO:BP	GO:0010876	Lipid localization	$1.29\times 10^{-25}$
XM	GO:CC	GO:0005789	Endoplasmic reticulum membrane	$6.16\times 10^{-23}$
OWG&CE	GO:MF	GO:0005102	Signaling receptor binding	$3.87\times 10^{-21}$
OWG&CE	GO:MF	GO:0042562	Hormone binding	$1.95\times 10^{-13}$
OWG&CE	GO:MF	GO:0120020	Cholesterol transfer activity	$8.205\times 10^{-11}$
OWG&CE	GO:BP	GO:0042592	Homeostati process	$4.17\times 10^{-64}$
OWG&CE	GO:BP	GO:1901700	Response to oxygen-containing compound	$2.025\times 10^{-62}$
OWG&CE	GO:BP	GO:1902652	Secondary alcohol metabolic process	$3.12\times 10^{-21}$
OWG&CE	GO:CC	GO:0005615	Extracellular space	$1.11\times 10^{-13}$
VMM&DRD	GO:MF	GO:0016491	Oxidoreductase activity	$7.25\times 10^{-21}$
VMM&DRD	GO:MF	GO:0019842	Vitamin binding	$1.65\times 10^{-18}$
VMM&DRD	GO:MF	GO:0046906	Tetrapyrrole binding	$7.76\times 10^{-11}$
VMM&DRD	GO:BP	GO:0006766	Vitamin metabolic process	$5.69\times 10^{-46}$
VMM&DRD	GO:BP	GO:0006575	Modified amino acid metabolic process	$6.58\times 10^{-32}$
VMM&DRD	GO:BP	GO:0006082	Organic acid metabolic process	$5.845\times 10^{-24}$
FA	GO:MF	GO:0005126	Cytokine receptor binding	$2.90\times 10^{-54}$
FA	GO:MF	GO:0140375	Immune receptor activity	$2.74\times 10^{-45}$
FA	GO:MF	GO:0019955	Cytokine binding	$4.60\times 10^{-31}$
FA	GO:BP	GO:0006955	Immune response	$4.41\times 10^{-174}$
FA	GO:BP	GO:0051767	Nitric oxide synthase biosynthetic process	$5.19\times 10^{-12}$
FA	GO:BP	GO:0070672	Response to interleukine-15	$5.89\times 10^{-11}$
FA	GO:CC	GO:0009897	External side of plasma membrane	$4.14\times 10^{-64}$
FA	GO:CC	GO:0005576	Extracellular region	$1.81\times 10^{-22}$
FA	GO:CC	GO:0045121	Membrane raft	$5.56\times 10^{-15}$
FI	GO:MF	GO:0042802	Identical protein binding	$3.07\times 10^{-16}$
FI	GO:BP	GO:0070887	Cellular response to chemical stimulus	$5.62\times 10^{-32}$
FI	GO:BP	GO:0042592	Homeostatic process	$2.28\times 10^{-29}$
FI	GO:BP	GO:0036297	Interstrand cross-link repair	$6.51\times 10^{-11}$
FI	GO:CC	GO:0043240	Fanconi anemia nuclear complex	$7.01\times 10^{-13}$
FI	GO:CC	GO:0098590	Plasma membrane region	$1.17\times 10^{-11}$
FI	GO:CC	GO:0031982	Vescicle	$1.19\times 10^{-11}$
