# Supplementary material for: Missense Variants in Nutrition-Related Genes: A Computational Study
Source: Int J Mol Sci. 2025 Oct 2;26(19):9619. doi: 10.3390/ijms26199619 (PMC12525056; doi:10.3390/ijms26199619)
Supplement: Supplementary file 1 [file Figure_S1.pdf › Figure_S1.pdf]

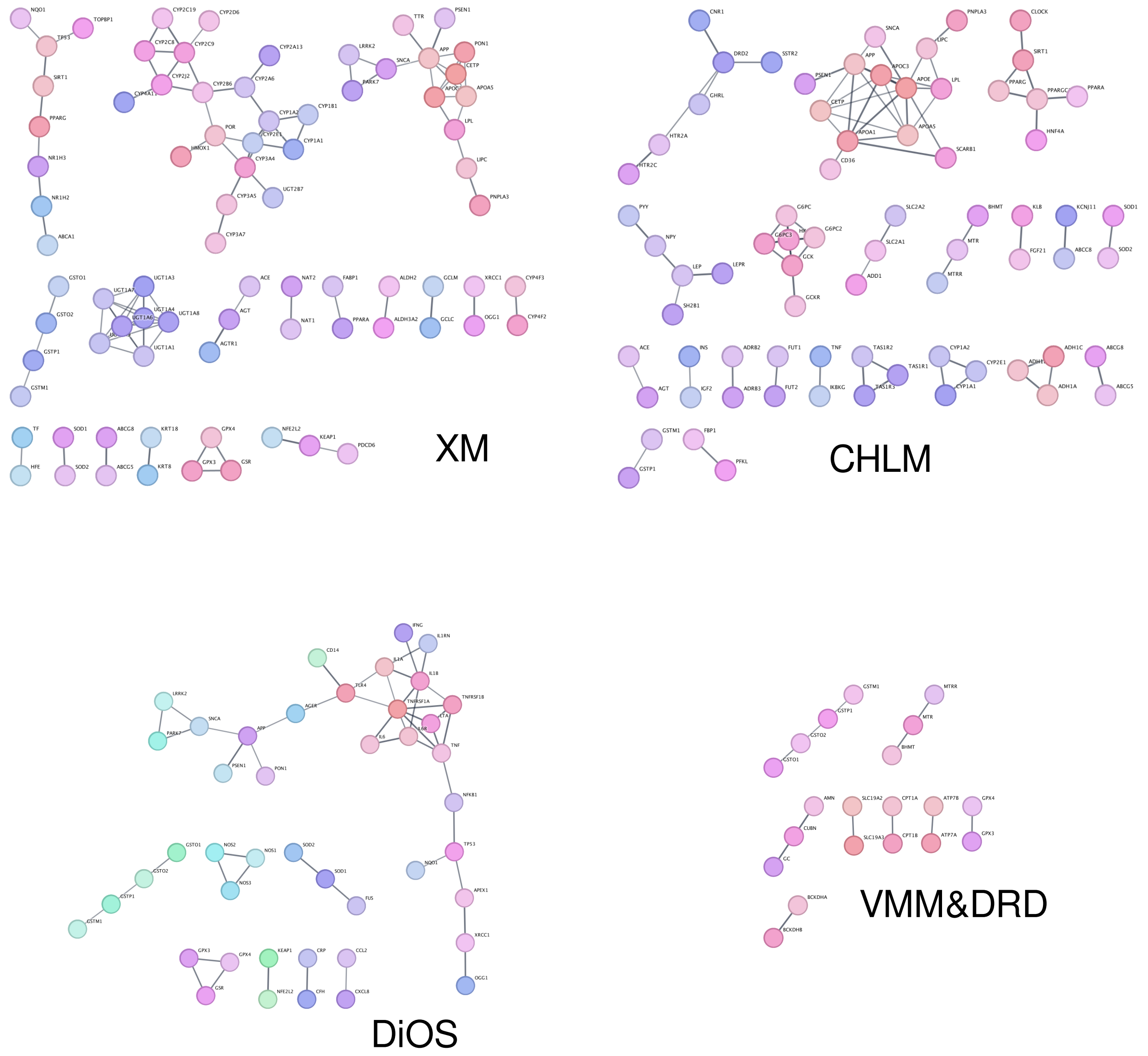

Supplementary Figure S1. Protein–protein interaction (PPI) networks for four nutrigenetic topics with limited gene set sizes after filtering out singletons (i.e. isolated genes): XM (Xenobiotics Metabolism), CHLM (Cardiovascular Health and Lipid Metabolism), DiOS (Diet-induced Oxidative Stress), and VMM&DRD (Vitamin and Micronutrients Metabolism and Deficiency-Related Diseases). Networks were generated using Cytoscape with high-confidence (score ¿ 0.7) physical interactions from STRINGdb. Due to the small number of connected nodes, CytoHubba-based hub protein analysis was not applied to these networks.
